# Identification and Structure Prediction of Human Septin-4 as a Biomarker for Diagnosis of Asthenozoospermic Infertile Patients—Critical Finding Toward Personalized Medicine

**DOI:** 10.3389/fmed.2021.723019

**Published:** 2021-12-03

**Authors:** A. S. Vickram, K. Anbarasu, Palanivelu Jeyanthi, G. Gulothungan, R. Nanmaran, S. Thanigaivel, T. B. Sridharan, Karunakaran Rohini

**Affiliations:** ^1^Department of Biotechnology, Saveetha School of Engineering (SSE), SIMATS, Chennai, India; ^2^Department of Bioinformatics, Saveetha School of Engineering (SSE), SIMATS, Chennai, India; ^3^Department of Biotechnology, Vel Tech Rangarajan Dr. Sagunthala R&D Institute of Science and Technology, Chennai, India; ^4^Department of Biomedical Engineering, Saveetha School of Engineering (SSE), SIMATS, Chennai, India; ^5^Department of Biotechnology, School of Bio Sciences and Technology, Vellore Institute of Technology (VIT), Vellore, India; ^6^Unit of Biochemistry, Faculty of Medicine, AIMST University, Bedong, Malaysia

**Keywords:** human semen, seminal plasma, motility associated protein, septin-4, *in-silico* characterization, molecular dynamics simulations

## Abstract

Semen parameters are been found as a key factor to evaluate the count and morphology in the given semen sample. The deep knowledge of male infertility will unravel with semen parameters correlated with molecular and biochemical parameters. The current research study is to identify the motility associated protein and its structure through the *in-silico* approach. Semen samples were collected and initial analysis including semen parameters was analyzed by using the World Health Organization protocol. Semen biochemical parameters, namely, seminal plasma protein concentration, fructose content, and glucosidase content were calculated and evaluated for correlation. Sodium dodecyl sulfate-polyacrylamide gel electrophoresis (SDS-PAGE) and matrix-assisted laser desorption/ionization-time of flight (MALDI-TOF) were carried out for identification of Septin-4 presence in the semen sample. Mascot search was done for protein conformation and *in*-*silico* characterization of Septin-4 by structural modeling in Iterative Threading Assembly Refinement (I-TASSER). Twenty-five nanoseconds molecular dynamics (MD) simulations results showed the stable nature of Septin-4 in the dynamic system. Overall, our results showed the presence of motility-associated protein in normospermia and control samples and not in the case of asthenospermia and oligoasthenospermia. Molecular techniques characterized the presence of Septin-4 and as a novel biomarker for infertility diagnosis.

## Introduction

Human infertility affects <15% of all couples, <6% of Indian couples. Among these, male partner contributes 40–50% of total infertility (1–3). This gave a clear picture of the contribution of males toward human infertility. Semen parameters, namely, spermatozoa concentration, sperm motility, morphology, etc. plays a major role and act as a deciding factor for fertility rate. So, the andrologists majorly focus on these issues primarily toward the diagnosis of male infertility (4, 5). For analyzing these factors, a multiple-omics approach is in need to diagnosis male infertility by having a strong focus on parameter analysis. Semen parameters were found to be only the primary trump card, with these, we can just tell about the count and morphology, wherein the deepest knowledge of male infertility will come only when semen parameters were correlated with many molecular and biochemical parameters (6). One such approach is proteomics of semen, correlating with motility-associated proteins. Motility is the major parameter analyzed during semen analysis, the cluster of proteins involved in giving mobility to the sperm cells when entered into the female reproductive system (7–11). Many potential biomarkers could be elucidated here (proteomic approach) which strengthens the diagnosing part (12). A biomarker is a marker derived from any biological substances which could be used to study, analyze, and compare various conditions and strategies. Biomarkers were non-invasive, with minimal side effects, and could be used for various diagnostics and therapeutics values (13–17). Currently, the basic andrology laboratory, various semen analysis parameters, endocrine research, and antisperm antibodies where assisting clinicians for diagnosis (18–20).

In addition, the proteomic approach will strengthen the patient-specific diagnosis and prognosis. Already we studied the role and influence of many proteins like Semenogelin II, prostasomes proteins, and epididymal proteins as markers for various diagnostic approaches. Septin is one of the flagellar proteins that produce the energy in the annular region and helps the sperm to move forward in the female reproductive tract (21–23). Septins are the major cytoskeletal protein with major and unique filament-forming capabilities (24, 25). Many mice model studies proved that the downregulated or missing septin family protein in ejaculated semen will fall under sick without mobility and thus producing the immotile sperms will not help further for natural conception (26). So far 14 different septin genes were identified since the first was 35 years back. Disruption of septin and its functions shows many abnormalities to humankind, namely, neoplasia, breast cancer, Parkinson's disease, neurogenerative disease, and human male infertility (27, 28). Each septin presence is important for other septins to do their functions properly. These septins will bind together to produce a higher order structure, to form a filament, membranes, or ring-like structure (29). The septin-rich part of sperm is the annulus, it is a submembranous ring that separates the middle and peripheral regions of the sperm flagella. The role of septin is still in debate whether it is an active GTPases or just as a guanosine triphosphate binding protein (30, 31). Septin gives much more energy and the ring structure gives the circulatory force that drives the sperm to move forward and not immotile in the female reproductive tract (32, 33).

The functions of septin start at spermatogenesis itself, during this time it helps in establishing the mitochondrial architecture and cytoskeleton to the annulus. The absence of Septin-4 and−12 in the sperm cell, lacking with the functions of mobility, midpiece damage, rounded sperm head, acrosomal defects, etc. (34, 35). Many studies revealed the insufficient energy for a sperm cell to move forward in the absence of septin proven by *in vitro* and *in vivo* mice models. The absence of septin in sperm cells is shown with lots of annuli and the connection between midpiece and head, this will misfunction the sperm and not able to fuse the ovum as it fails the forward motility (36, 37). The functions of septin in male fertility were more, but still, the mechanism of understating these family proteins was very tough, and correlating with male infertility diagnosis could be elucidated further (38, 39). Due to the lack of experimental structure of human Septin-4, the structural prediction methods using *in-silico* characterization will help in elucidating the structure–function relationship at the molecular level.

## Materials and Methods

### Semen Sample Collection

Semen samples were collected from the patients who visited Bangalore Assisted Conception Center, Bangalore, Karnataka at the Andrology lab. The samples were collected from them in a wide mounted, sterile, non-toxic plastic ware, they have been provided with a neat room to collect the samples. The method followed was 7 days abstinence time and masturbation technique. Strictly the abstinence time was asked with them as it influences the results in a great manner. The patients were provided with all necessary infrastructures for collection as this also influence the results. Once, the collection was over, the patient details, namely, the name, hospital number, andrology number, abstinence time, method of collection, smoking habits, alcohol habits, last visit date, last collection date, age, and region were asked for and observed. The sample container was marked with a patient number, hospital number for further processing (40).

### Ethical Consent

Ethical clearance was done for this work to carry on human semen samples. Informed consent was also obtained from the patients in their own language. The patients were explained with the motive of this work and only after semen analysis report were ready, and then the remaining samples were utilized for this work.

### Semen Analysis Report Preparation

Soon after the arrival of samples from the patients to andrologists, a semen analysis report was prepared. World Health Organization (41) procedure was strictly followed to prepare the report. Computer-assisted semen analysis, Germany made, was used to compute the number of spermatozoa, motility, morphology, etc. (42).

### Categorization of Semen Samples

Semen samples were segregated into groups by prepared semen analysis report; the categories were asthenozoospermia, oligozoospermia, normozoospermia, and healthy volunteers or controls. The segregation was done purely by using semen parameter values and semen analysis reports (42).

### Statistical Analysis

We have used Graphpad prism (GraphPad Software, USA), version 5.1 for this research statistical data. Values were mentioned with mean ± standard error of the mean for experiments repeated (43).

### Separation of Spermatozoa and Seminal Plasma for Biochemical and Molecular Analysis

For this research, after semen sample analysis, samples were collected according to the standard protocol followed by WHO and as per Rao et al. (44).

### Spermatozoa Disruption for Obtaining the Intracellular Protein Content

Spermatozoa separated from seminal plasma; sperm pellets were suspended which was supplied with buffers with various detergents. The standard protocol is followed for spermatozoa disruption (42).

### Protein Estimation

Protein estimation was done on each fraction of seminal plasma and spermatozoa with the standard protocol followed by standard protocol (45).

### Fructose Content Estimation

Fructose content in each sample was evaluated with the standard protocol given by WHO (41), with some modifications done (46).

### Enzyme α-Glucosidase Estimation

α-Glucosidase estimation in each sample was evaluated with the standard protocol given by WHO (41), with some modifications done (47).

### Estimation of Trace Element Zn

Zinc (Zn) plays a major role in human male fertility. Estimation of Zn was done with standard protocol by using atomic absorption spectroscopy and followed standard protocol (48). Trace element concentrations were estimated using the standard curve.

### Identification of Septin-4 Protein in Spermatozoa

The centrifuged and ultrasonicated samples were used to identify the fertility-associated protein in spermatozoa (Septin-4); intracellular proteins isolated from different semen samples' categories (asthenozoospermia, oligospermia, normospermia, and control) were subjected to SDS-PAGE analysis. The silver stating protocol was used to stain the gel. To the extend, the protein band which was differentially expressed (downregulated) in the asthenozoospermia category was subjected to matrix-assisted laser desorption/ionization- time of flight- mass spectrometry (MALDI-TOF-MS) analysis and then Mascot search for identification of the protein.

The differentially expressed band from the gel was excised and dehydrated with a minimum of 50% 50 mM ammonium bicarbonate and 50% acetonitrile. Then follows the standard protocol overnight. Voyager-DE STR instrument (PerSeptive Biosystems, Inc., USA) in linear mode was used to acquire MALDI-TOF-MS spectra. Positive ions accelerated to 20 V were calculated. Both matrix and sample were dissolved in milliQ water and equal ratios of matrix and sample were mixed and spotted onto MALDI plate for analysis.

### *In-silico* Characterization

In addition to the wet-lab experiments, the *in-silico* structural analysis was evaluated for human Septin-4. The primary analysis based on the Swissprot database screen proved Septin-4 consists of 478 amino acids (Uniprot/Swissprot id: O43236). Septin-4 consists of eight isoforms and isoform 1 (identifier: O43236-1) was selected for the analysis consisting of molecular weight 55,098 Daltons (55 KDa). From the structural database screening, the absence of an experimental 3D structure of Septin-4 was identified. The *in-silico* structural modeling of Septin-4 was performed using the Iterative Threading Assembly Refinement (I-Tasser) server (49). Iterative Threading Assembly Refinement is a fully automated 3D structural prediction of protein server based on the threading/fold recognition methodology. It ranked no. 1 among the structural prediction server evaluated by a critical assessment of structure prediction (CASP14 experiment in 2020) and also ranked top for the function prediction (CASP9). The server chooses the suitable structural templates from database protein data bank (PDB) by a multiple-threading approach called local meta-threading server (LOMETS) and protein models constructed by iterative template-based fragment assembly simulations. The prediction is mainly based on critical parameters like C-score, TM score, and root mean square deviation (RMSD). C-score, a scoring function mainly based on the theoretical concepts were also done. C-score with a range of [−5, 2] signifies the higher value confirmed the protein model with the confidence level. The output showed the five best protein models based on optimal C-score, TM-score, RMSD, and SD.

### Molecular Dynamics Simulation

Molecular dynamics (MD) simulations study on human Septin-4 was carried out using GROMACS 5.0 package (David van der Spoel, Sweden) (50). Simple point charge (SPC21) water molecules of 0.9 nm were used for the solvation of protein models in the simulation box. The neutralization of the system was obtained by adding six sodium ions to replace the initial SPC water molecule in all directions. Energy minimization of all systems was carried out by steepest descent energy minimization with tolerance limit 100 kJ/mol and GROMOS96 43a1 force field was used for the simulations of protein (51). A cutoff of 14 Å for van der Waals interactions and 12 Å for electrostatic interactions was used for the process. Electrostatic interactions were computed using the particle mesh Ewald method. The LINCS algorithm was used to constrain all bond lengths and the SETTLE algorithm was applied to constrain the geometry of water molecules in the system. The energy minimization was done in two equilibration phases, number of particles, volume, and temperature (NVT) ensemble with a constant temperature of 300 K and with a coupling constant of 0.1 ps for duration 100 ps, and number of particles, pressure, and temperature (NPT) ensemble with a constant pressure of 1 bar was employed with a coupling constant of 5 ps for duration 100 ps. For both ensembles of equilibration, the coupling scheme of Berendsen was employed. Finally, the systems were subjected to production MD simulation for 25 ns run. MD trajectories of human Septin-4 were analyzed by GROMACS utilities. The analysis included RMSD, solvent accessible surface, the radius of gyration (Rg), and principal component analysis (PCA). The stability analysis was performed by using utilities like g_ rms, g_ sas, g_ gyrate, g_covar, and g_anaeig, respectively. Principal component analysis describes a correlated motion of the protein obtained from the mass-weighted Cα-covariance matrix. The functionally relevant motion of the protein can be computed by the collective displacement of domains called essential dynamics. To detect the collective motion mutant trajectories were subjected to PCA. The resulting covariance matrix describes the concerted coordinate motions.

In this study, the first and second Cartesian principal components are considered reaction coordinates derived from PCA.

## Results

The first step was to categorize the semen samples based on the World health organization values, this was done by using several semen samples, and each value and its error mean was the final mark. Based on the semen analysis report oligospermia (*N* = 18) meant for less count than normal, asthenospermia (less motility *N* = 24) than normal, normospermia (normal as per WHO *N* = 15), oligoasthenospermia (both less count and motility *N* = 12), and healthy volunteer (control *N* = 8). The semen parameter values were tabulated in Supplementary Table 1. The results suggested that there exists a potential statistical difference exist between oligospermia and asthenospermia in the case of motility parameter. As this work will further correlate only the motility issues, the results we majorly focused on only motility issues.

Once the semen analysis report and categorization of samples were done, immediately the samples were kept in liquid nitrogen preservation. Once the need, the samples were centrifuged for separation of seminal plasma and spermatozoa. Important biochemical parameters were analyzed. The total protein content was done for both seminal plasma and spermatozoa, fructose content was estimated in seminal plasma, α-glucosidase estimation was also done in seminal plasma for all samples in all categories, and Zn content was evaluated in the same way. All these are very essential biochemical parameters that need to be evaluated for proper correlation with molecular markers during diagnosis. All these biochemical values for different categories of semen samples were tabulated in Supplementary Table 2.

**Figure 1:**
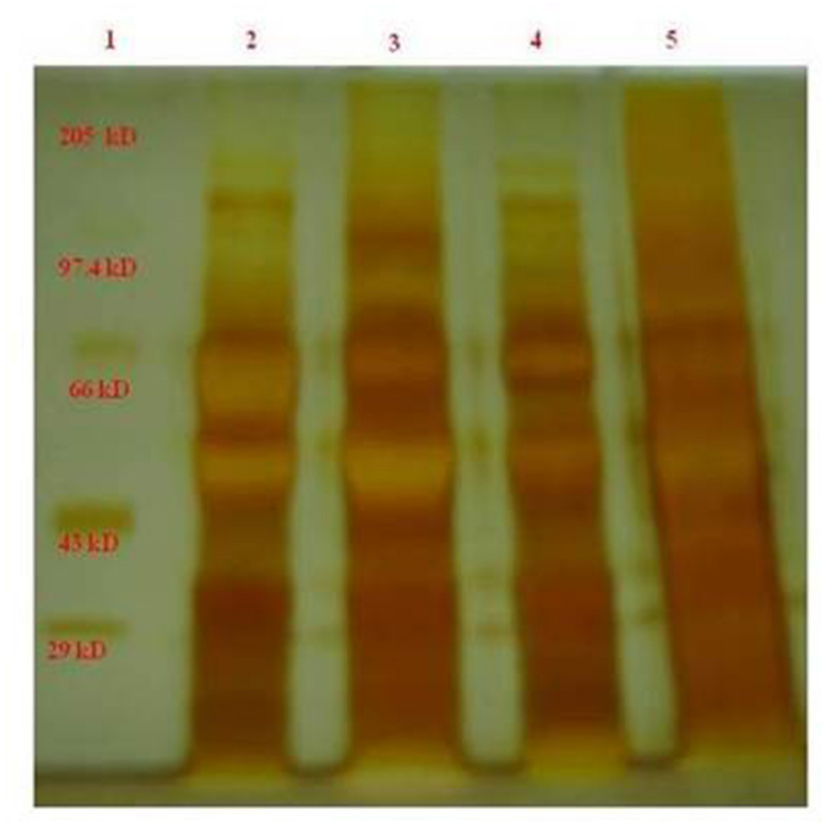
The band around 55 kDa was less expressed in the case of asthenospermia, but present in the case of oligospermia, normospermia, and healthy volunteers. We guessed the importance of missed 55-kDa protein and further we want to investigate on this protein. GelAnalyzer was used to analyze this 1D SDS PAGE bands and all the interpretation has been done by the standard protocol. 1, marker standard; 2, Normospermia; 3, healthy volunteer; 4, Asthenospermia; 5, Oligoasthenospermia.

Protein content was already evaluated through Lowry's method. After centrifugation, sodium dodecyl sulfate-polyacrylamide gel electrophoresis (SDS-PAGE) was done for different infertile categories as mentioned earlier in the methodology section. The developed silver-stained protein SDS-PAGE was depicted in Figure 1. Almost eight bands were found to be visible in the SDS-PAGE, with a maximum of bands existing in the case of 50 and 110 kDa proteins. The band around 55 kDa was missing in the case of asthenospermia, but present in the case of oligospermia, normospermia, and healthy volunteers. We guessed the importance of missed 55-kDa protein and further, we want to investigate this protein. The missed protein was isolated from normospermia and healthy volunteers and then MALDI-TOF analysis was done for eight samples to access the similarity in the results. Also, a Mascot search was done by using the MALDI-TOF results. The missing protein in asthenospermia was identified as Septin-4. It has already been evidenced that this protein had played a major role in Alzheimer's disease, male infertility, and Down syndrome. The role of Septin-4 in male infertility is enormous and more molecular work is in need for the prediction of the pathway mechanism behind male infertility. The correlation of motility and its implications with male infertility diagnosis is the key to success.

### Mascot Search and Its Implications

The date got through *m*/*Z* values were analyzed for each sample was searched in mascot MALDI-TOF-MS ions search. The database used as SwissProt, humans as chosen for taxonomy and enzyme as trypsin in the search tool. The parameters used for searching the protein of interest through mascot search were tabulated in Table 1. We looked for a maximum of hits and were obtained against the Septin-4 protein. The functions of the query protein were reviewed in Swissprot and involves in male infertility if downregulated in certain patients. Database search was performed in PDB and observed that the absence of experimental structure of human Septin-4. The *in-silico* approach has been used to predict the structure of protein for further research studies.

**Table 1:** Mascot Search for the identified protein by MALDI TOF and its parameter search.

**Variable modification**	**Protein**	**Fixed modification**	**Sequence coverage (%)**	**Significance score**
Carbamidomethyl (C)	Septin 4 (*Homo sapiens*)	Carbamidomethyl (N-term)	91	85
Carbamidomethyl (C) Carbamyl (K)	Septin family (Mice)	Carbamidomethyl (N-term) Carbamyl (N-Term)	85	57
Carbamidomethyl (C) Carbamyl (K)	Semenogelin II (*Homo sapiens*)	Carbamidomethyl (N-term) Carbamyl (N-Term)	80	52

### Structure Prediction

**Figure 2:**
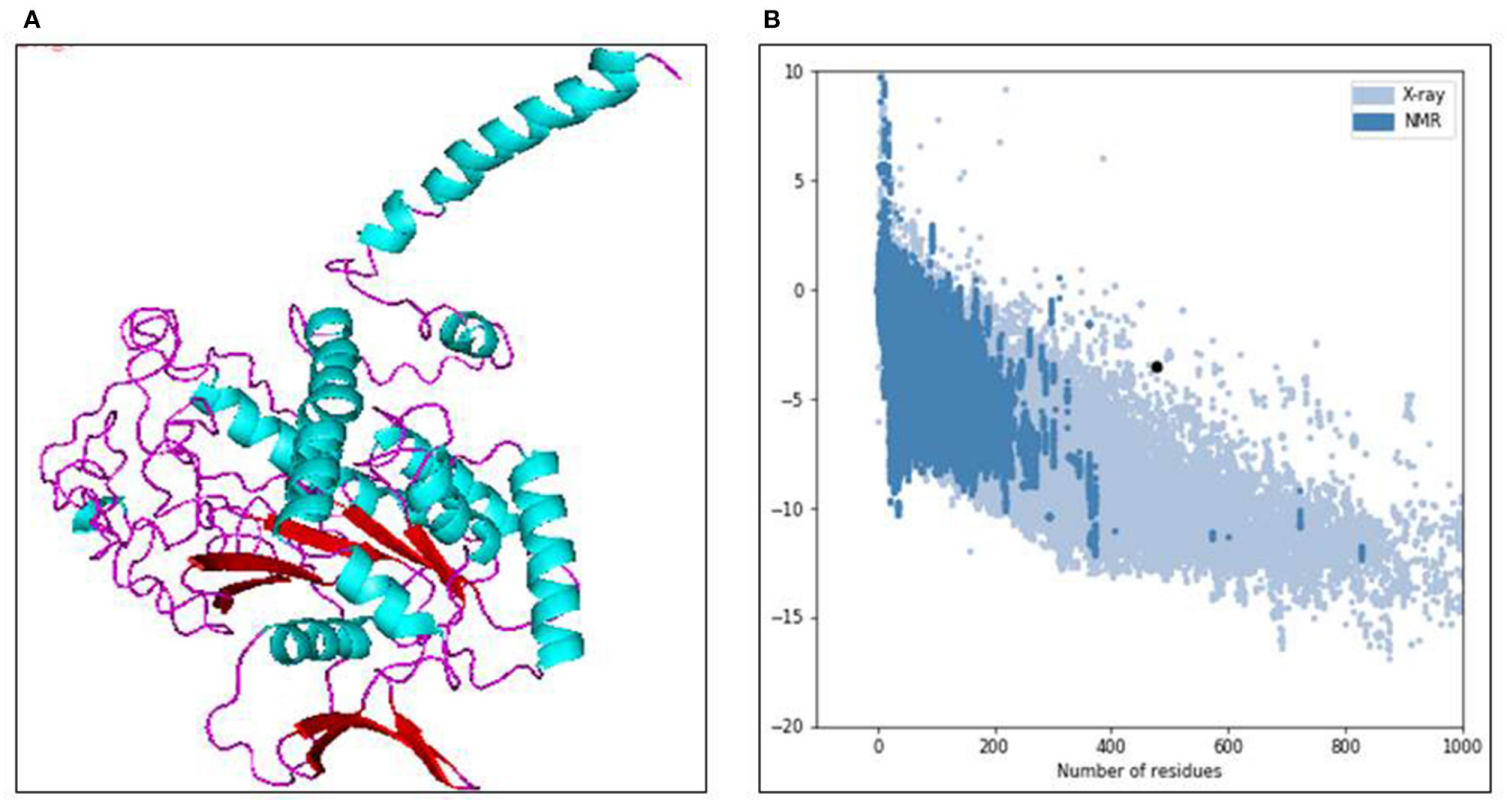
**(A)** 3D structure model of human septin 4 visualized in PyMOL. **(B)** Protein quality check from ProSA server.

Using the *in-silico* structural study on human Septin-4, the 3D structural model was predicted from the I-Tasser server. Out of five models, the model with the least C-score −3.19 was selected as the best structure of Septin-4. The other parameters also supported the model with an estimated TM score of 0.36 ± 0.12 and an estimated RMSD of 15.2 ± 3.5Å. The threading/fold recognition method screened the structure of the GTPase domain of human Septin-12 (PDB code: 6MQ9) as the template for the Septin-4 modeling. The Septin-4 model falls under the structural classification of alpha + beta, the architecture of the three-layer (α*βα*) sandwich, and the topology of the Rossmann fold, and is visualized in PyMol in Figure 2A. The major molecular function of the septin family was catalytic activity, GTPase activity, hydrolase activity, protein binding, lipid binding, and protein dimerization activity. The quality of the model was deciphered by the ProSA server and results showed the *Z*-score of −3.5 that related to experimental structures in Figure 2B. The above predicted human Septin-4 structural model can be used for further annotation studies related to male infertility mechanisms.

### Molecular Dynamics Simulations

**Figure 3:**
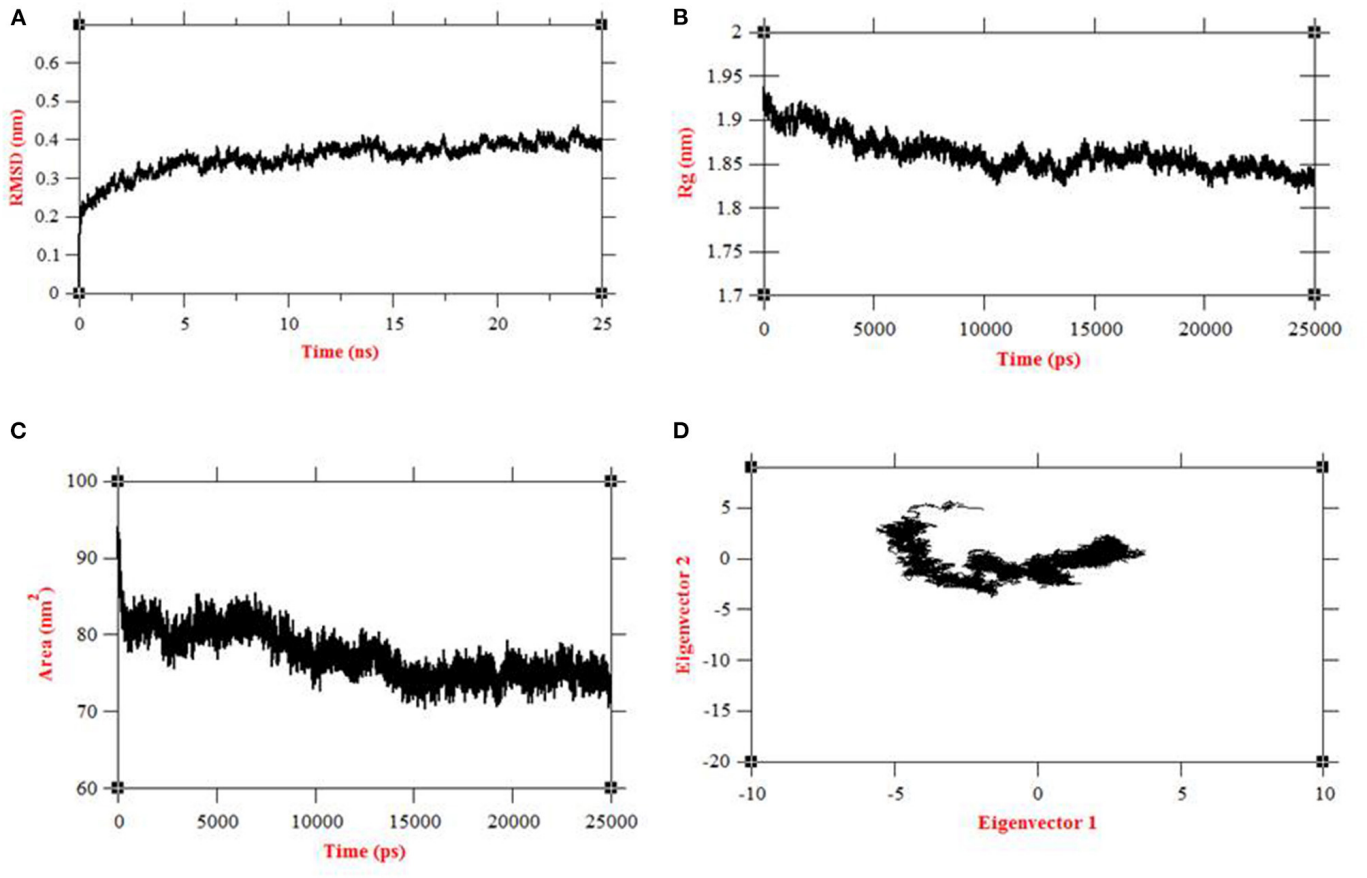
Molecular dynamics simulations at 25ns of human septin 4 model **(A)** RMSD plot, **(B)** Rg plot, **(C)** SASA plot, **(D)** PCA plot.

The convergence of the protein system during simulations was measured by RMSD of all Cα atoms from the initial structure. The initial equilibration of the native structure of human Septin-4 was done in 5 ns. After the equilibration phase, the structure of Septin-4 showed an RMSD range in 0.3–0.4 nm during 25 ns simulations (Figure 3A). The structure was well-converged and confirmed the protein stability of Septin-4 at end of simulations and structure with a stable trajectory in the dynamic system. Radius of gyration was the property of the overall dimension of protein during simulations. The Rg is termed as a measure of mass-weighted root mean square distance of all atoms from the center of mass. Radius of gyration of Septin-4 native structure started with 1.92 nm but gradually decrease to equilibrate with 1.85 nm (Figure 3B). Thus, the overall protein folding pattern of human Septin-4 protein was observed. A solvent-accessible surface (SASA) plot was constructed and results showed the accessibility area around 75–80 nm^2^ confirmed the behavior of the hydrophilic and hydrophobic residues in Septin-4 (Figure 3C). Principal component analysis was performed based on two steps. In the first step, the covariance matrix was constructed and diagonalized based on Cα atoms using g_covar and trace value of 5.52816 nm^2^. The eigenvectors and corresponding eigenvalues were evaluated from the covariance matrix using the motion of protein at the atom level. Then PCA was done using g_anaeig with the projection of the first two eigenvectors (eigenvector 1 vs. eigenvector 2) and the maximum motion extracted from the production run of 20 ns. The local motion of the PCA plot showed the overall motion of human Septin-4 in the dynamic system related to eigenvector 1 vs. eigenvector 2. The cluster was more compact and deciphered the motion of protein with covariance matrix (Figure 3D).

## Discussion

Homozygous Septin-4 (Human semen Septin-4) deletion or its downexpression was shown to have a complete or partial defect in the structure of the sperm flagellum; this means it helps a lot for the forward motility (52, 53). In our results also, the Septin-4 absent or less expressed yield with less motility and especially with forward type. The defect in the flagella or neck region always yields these types of results (54, 55). Other researchers worked with Septin-4-null sperm or flagella modified with no annulus, this structure has been replaced by thin segment missing cortical material, acts like an abnormal-flagella conferring a hairpin-like structure (56–58). Two major hypothetical utilities have long been ascribed to the annulus of the spermatozoa: one is a diffusion barrier function; it is a very essential function for the fertilization, detaining proteins to various compartments of the sperm tail to the neck (59, 60). The second one is might be on morphological planner function given guidance to the growth of the flagellum and the association of the mitochondria along the axoneme. Both of these mechanisms were found to be failed in the case of Septin-4 null sperm. Morphology of human sperm annulus/flagellum has been known for a long time, but the mechanism by which it is correlating is poorly studied (56, 61). Sperm flagella biogenesis, the biochemical composition of the sperm tail to neck, and its functions remained as same in the case of rigorous research. For the last decade, septins have appeared and been explored as constitutive components of the annulus/flagella of spermatozoa and persuasive evidence has been evidenced by many researchers and suggest that a very stable septin complex/Septin-4 is the prerequisite for morphological differentiation of the sperm tail, neck and with an important mechanism of diffusion barrier function (56). Although current evidence suggests that septins bind to the plasma membrane via interaction with phosphoinositides, our previous research with prostasomes suggest that the Zn present on prostasomes may transfer the essentials of needed motility factors and phospholipids for proper movement (62), this achieved through the fusion process of prostasomes and spermatozoa by means of protein dependent or pH dependent (63, 64). This finding suggests that binding to integral membrane proteins could also be involved. Moreover, the advance of *in-silico* studies deciphered the structural annotation of human Septin-4 that can be used to understand the role of septin in male infertility. Molecular modeling is the current best method used in the 3D structure prediction of key protein/enzymes/drug targets in proteomics. From the model structure, the major mechanism of Septin-4 has been studied using the structural arrangements of helix and sheets. The structure–function relationship is highly critical in the research area of male infertility, as very few 3D experimental structures are available. Also, advancements in MDs simulation deciphered the behavior of novel biomarker protein Septin-4 in the all-atom dynamics. *In-silico* finding acts as a critical point that can initiate various structure-function studies on human Septin-4 toward male infertility mechanism and pharmacology aspects.

## Conclusion

Septins are the most important constituents of the annulus in spermatozoa, a submembranous ring that disconnects the middle and primary pieces of spermatozoa. This is believed to be an important protein Septin-4 that plays a major role in motility and its absence may be associated with asthenospermia. Many researchers previously reported its essential role in spermatogenesis and reproduction in animal models. Till now many researchers worked with labeling techniques and identified the importance of Septin-4 in the case of male infertility. In this current research work, we elucidated and identified the presence of Septin-4 in normal healthy sperm samples and its absence or less expression in the case of other infertile groups especially in the case of motility-related issues. The importance of Septin-4 in male fertility was proved with 3D structural modeling from *in-silico* characterization and MDs simulation confirmed the role of stable Septin-4 in the dynamic system. Less expression was found exclusively in infertile patients when compared to fertile patients. Further research on Septin-4 with structural studies may be used to explore more on the mechanism and its role in spermatogenesis and human infertility. Hence, our findings concluded that Septin-4 was a novel biomarker for male infertility and can be used for diagnosis and pharmacology purposes.

## Data Availability Statement

The raw data supporting the conclusions of this article will be made available by the authors, without undue reservation.

## Ethics Statement

The studies involving human participants were reviewed and approved by Dr. Radha Saraswathy, Member Secretary-UHEC, Senior Professor, SBST, VIT, Vellore, Tamilnadu, India. The patients/participants provided their written informed consent to participate in this study.

## Author Contributions

ASV involved in conceptualization, design protocol, performed experimental analysis, and wrote the manuscript on *in-vitro* studies section. KA involved in conceptualization, protocol design, experimental analysis, and wrote the manuscript on *in-silico* studies section. PJ, GG, RN, ST, and TS were involved in data validation, critical assessment, and edited the manuscript. KR involved in supervision of the research work and edited the final manuscript. All authors contributed to the article and approved the submitted version.

## Conflict of Interest

The authors declare that the research was conducted in the absence of any commercial or financial relationships that could be construed as a potential conflict of interest.

## Publisher's Note

All claims expressed in this article are solely those of the authors and do not necessarily represent those of their affiliated organizations, or those of the publisher, the editors and the reviewers. Any product that may be evaluated in this article, or claim that may be made by its manufacturer, is not guaranteed or endorsed by the publisher.
