# Supplementary material for: Identification and Structure Prediction of Human Septin-4 as a Biomarker for Diagnosis of Asthenozoospermic Infertile Patients—Critical Finding Toward Personalized Medicine
Source: Front Med (Lausanne). 2021 Dec 3;8:723019. doi: 10.3389/fmed.2021.723019 (PMC8677696; doi:10.3389/fmed.2021.723019)
Supplement: Supplementary file 1 [file Data_Sheet_1.docx]

**Supplementary Table 1:** Comparison of Semen parameter as per World health organization (WHO)

| **Semen category** | **Volume**  **(ml)** | **pH** | **Sperm concentration (millions/ml)** | **Total motility (%)** | **Rapid progressive motility (%)** | **Normal morphology (%)** |
| --- | --- | --- | --- | --- | --- | --- |
| Oligoasthenospermia (N=12) | 3.4±0.4 | 7.8±0.1 | 5.6±0.5 | 6.7±1.2 | 3.45±0.5 | 18.5±3.5 |
| Asthenospermia (N=24) | 2.7±0.5 | 7.7±0.0 | 27.7±2.9 | 8.2±1.5 | 3.2±0.6 | 12.4±2.4 |
| Oligospermia  (N=18) | 2.8±0.5 | 7.7±0.1 | 6.5±0.3 | 18.8±3.5 | 23.8±3.9 | 15.8±3.5 |
| Normospermia (N=15) | 3.4±0.7 | 7.7±0.1 | 85.4±12.4 | 62.4±10.3 | 35.2±6.5 | 28.5±1.6 |
| Control (N=08) | 3.7±0.5 | 7.7±0.0 | 94.1±12.8 | 66.5±5.7 | 30.5±4.02 | 25.5±2.1 |

**Supplementary Table 2:** Comparison of Biochemical parameters for diagnosis of human male infertility for categorization as per World health organization Protocol

| **Semen category** | **Protein concentration (mg/ml) Spermatozoa** | **Fructose concentration**  **(μmol/ejaculate)** | **α-Glucosidase estimation**  **(mU/ejaculate)** | **Zn concentration**  **(mg/ml)** |
| --- | --- | --- | --- | --- |
| Oligoasthenospermia (N=12) | 2.55 ± 0.45 | 6.54±1.2 | 9.26±0.48 | 0.18±0.05 |
| Asthenospermia (N=24) | 3.46 ± 0.88 | 7.45±0.25 | 12.12±0.16 | 0.22±0.08 |
| Oligospermia  (N=18) | 2.1± 0.11 | 9.20± 0.65 | 14.22±0.67 | 0.18±0.01 |
| Normospermia (N=15) | 5.36 ± 0.91 | 14.32±0.45 | 25.44±1.25 | 0.77±0.12 |
| Control (N=08) | 6.99± 0.77 | 22.34±4.14 | 28.66±1.12 | 0.85±0.04 |
